# Diamond Technique: A Triple Treatment for Neck and Décolletage Rejuvenation

**DOI:** 10.1111/jocd.16667

**Published:** 2024-12-04

**Authors:** Matheus dos Santos Teodoro, João Henrique da Fonseca Armada Barros, Ana Lúcia Gonzaga da Cunha

**Affiliations:** ^1^ Instituto Corpo e Pele Belo Horizonte Minas Gerais Brazil; ^2^ Clínica Skinline Santo André São Paulo Brazil

**Keywords:** biostimulatory treatment, calcium hydroxylapatite, collagen, dermis, neck, neocollagenesis, rejuvenation

## Abstract

**Background:**

In the pursuit of integral beauty and a harmonious overall appearance, individuals are increasingly seeking cosmetic treatments for both facial and extrafacial regions. Among these areas, the neck and décolleté have become focal points for care due to their visibility and susceptibility to postsurgical scarring and dyschromia compared with the face. Environmental stressors and sun exposure contribute to aging signs in these areas, prompting a demand for effective, minimally invasive procedures with reduced downtime and minimal risks. Biostimulators, hyaluronic acid, laser therapy, microfocused ultrasound and neurotoxins have become key players in addressing these concerns.

**Aims:**

This manuscript introduces the Diamond technique, which uses a diamond‐shaped acetate device to perform a minimally invasive protocol combining calcium hydroxyapatite, hyaluronic acid, and incobotulinumtoxinA for neck and décolleté rejuvenation.

**Patients/Methods:**

The technique involves two steps: solution preparation and device‐guided injections. The diamond‐shaped device was developed to guide the injections, enhancing precision and reproducibility and facilitating medical training. The device's strategic markings aid in solution application, ensuring a multidimensional approach to rejuvenation. Four female patients, aged between 55 and 63 years were used to demonstrate the technique, with two categorized as grade II and two as grade III according to the Merz scale of neck laxity at rest.

**Results:**

The four examples demonstrate improvements in skin laxity, wrinkles, and overall skin quality. Two participants reported “good results,” and two reported results “beyond expectations.” The Diamond technique's potential lies in its ability to provide predictable outcomes, individualized treatments, and patient satisfaction.

**Conclusion:**

While the authors assert the technique's safety and efficacy, the need for controlled studies is crucial. Rigorous, long‐term studies are warranted to validate its effectiveness and safety compared with the existing approaches.

## 1 Introduction

With the growing awareness of integral beauty and the desire for a harmonious overall appearance, people are seeking cosmetic treatments that target not only the face but also the highly visible extrafacial regions. Among these body sites, the neck and décolleté are frequently requested for care, especially minimally invasive procedures, as these areas are prone to scars and dyschromia after surgical or ablative procedures [1, 2].

The neck is a multifaceted region with distinct layers. The superficial layer includes the skin, a thin subcutaneous tissue, and platysma muscle. Beneath these, there are deeper structures, such as the sternocleidomastoid muscle, internal and external jugular veins, carotid arteries, the thyroid gland, and an intricate net of muscles associated with deglutition and phonation. In particular, the platysma muscle, extending from the chest and shoulders to the face and jawline, is essential for facial expressions. The décolletage refers to the upper chest area, including the clavicles and sternum. The skin in this region is thinner and more prone to photodamage and wrinkling due to sun exposure. The décolletage area is supported by the pectoral muscles and various ligaments [3, 4].

Neck and décolleté areas are commonly exposed to the sun and environmental stressors, which lead to signs of aging, including sagging skin, muscle laxity, wrinkles, spots, and loss of elasticity [3, 5]. Consequently, practitioners are adapting their approaches and utilizing innovative methods (less invasive, painless procedures with quick recovery periods and lower risks of procedure‐related complications) to meet the rising demand for neck and décolleté rejuvenation. Biostimulators, hyaluronic acid, laser therapy, microfocused ultrasound, and neurotoxins are the leading methods for minimally invasive rejuvenation of these areas [6, 7, 8, 9].

Calcium hydroxylapatite (CaHA) is a resorbable regenerative biostimulator. This formulation is used to stimulate skin collagen and elastin, angiogenesis, and dermal cell proliferation, improving the skin's biomechanical properties. The product can be diluted for injection in the face or in other body areas, such as the arms, abdomen, or neck, to regenerate skin architecture, increasing the strength and elasticity of the dermis, although regulatory approvals for these uses are scarce [6, 10, 11, 12, 13, 14].

Hyaluronic acid (HA) is used as a dermal filler to restore volume, smoothen wrinkles, and enhance skin hydration. When HA is applied to the neck and décolletage, it can help lift sagging skin, reduce the appearance of horizontal lines and wrinkles, and improve overall skin quality. By replenishing lost volume, HA injections create a more youthful and rejuvenated appearance [15, 16].

IncobotulinumtoxinA (INCO) is a highly purified botulinum toxin without other unnecessary clostridial proteins. Treatments for neck and décolleté rejuvenation have been reported to reduce the appearance of horizontal lines and soften the platysmal bands; in the décolletage, INCO can smooth wrinkles caused by sun damage and natural aging, providing a rejuvenated appearance [17, 18].

The association of these techniques potentializes the aesthetic results; nevertheless, compared with facial techniques, there are relatively few published strategies for neck and décolleté rejuvenation [8, 19, 20]. Therefore, this manuscript aimed to present a diamond‐shaped acetate device to perform a minimally invasive protocol using three products (CaHA, HA, and INCO) in the neck and décolletage regions (Diamond technique) to promote a multidimensional approach to rejuvenation. The goal of the procedure was to maximize the aesthetic improvement of the area with minimal adverse effects.

## 2 Methods

**FIGURE 1:**
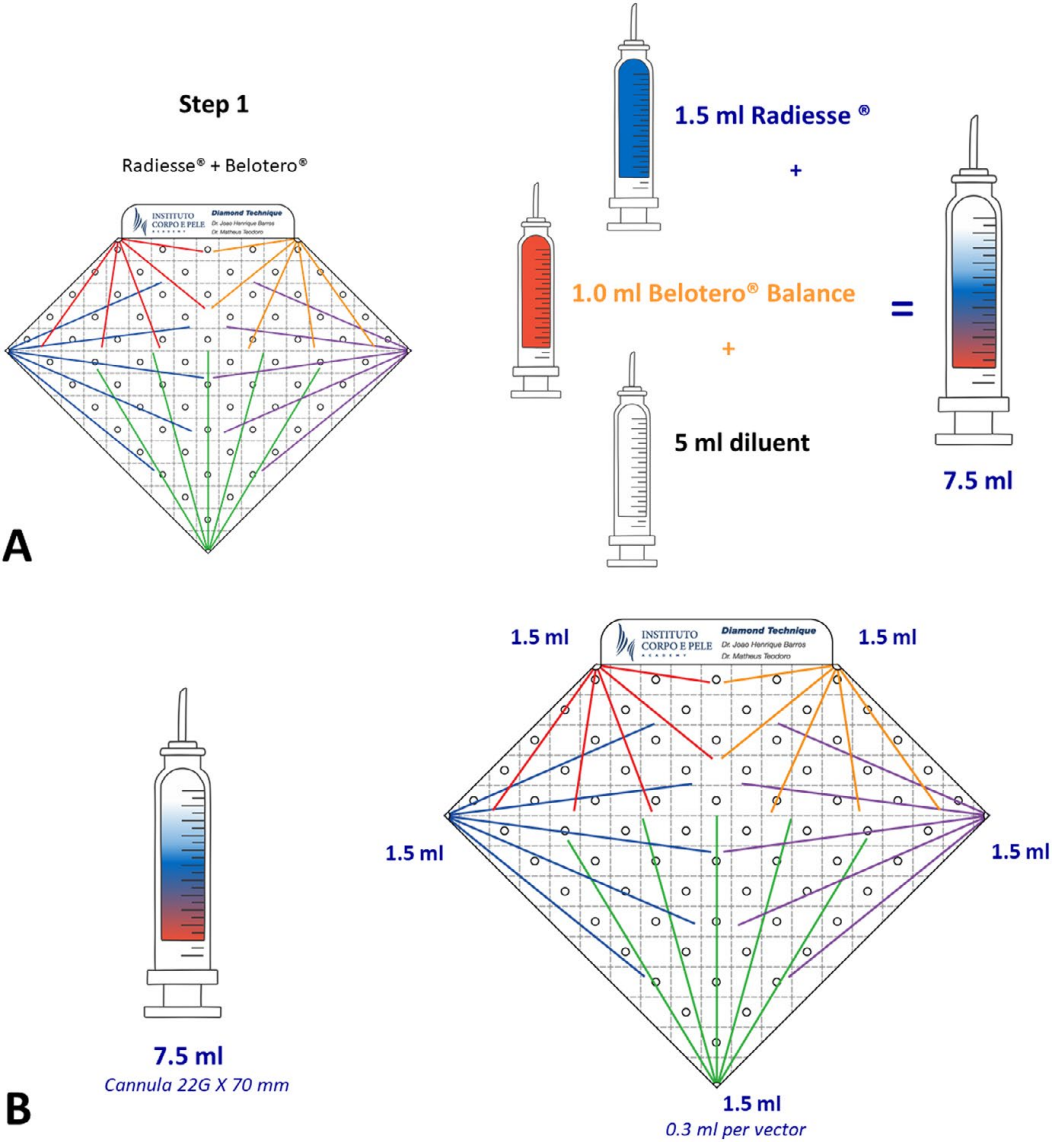
(A) Schematic representation of the mixing solution containing diluted CaHA and HA for the “fan mode” application of subdermal retroinjections (0.3 mL per vector) guided by the five vector groups (orange, red, green, purple, and blue), starting from the five vertices of the “diamond” on the neck (upside down/mirror image) and the décolleté. (B) Representation of the solution volumes for subdermal retroinjection from the five vertices of the “diamond” in five vectors: 0.3 mL each.

The Diamond technique can be divided into two steps: the preparation of the solutions and the use of a diamond‐shaped acetate device to guide the skin injections. The device is a pentagonal acetate plate (18 × 14 cm = 200 cm^2^) with 71 orifices. The device must undergo a 70% alcohol‐based antiseptic procedure before each treatment (Figure 1).

The first step involves mixing a solution containing CaHA and HA in the same syringe. A syringe containing 1.5 mL of CaHA (Radiesse, Merz Aesthetics) is combined with 1 mL of HA (Belotero Balance, Merz Aesthetics) and 5 mL of a solution composed of 4 mL of saline (NaCl 0.9%) and 1 mL of 2% lidocaine (Xylestesin, Cristália, Brazil), making a total volume of 7.5 mL (Figure 1). These products were mixed with two sterile polypropylene 10‐mL syringes using a female‐to‐female Luer‐lock connector. Mixing was performed with 10 strokes, and each mixing stroke consisted of one complete compression of the 10‐mL syringe plunger followed by another complete compression of the counterpart syringe [21].

**FIGURE 2:**
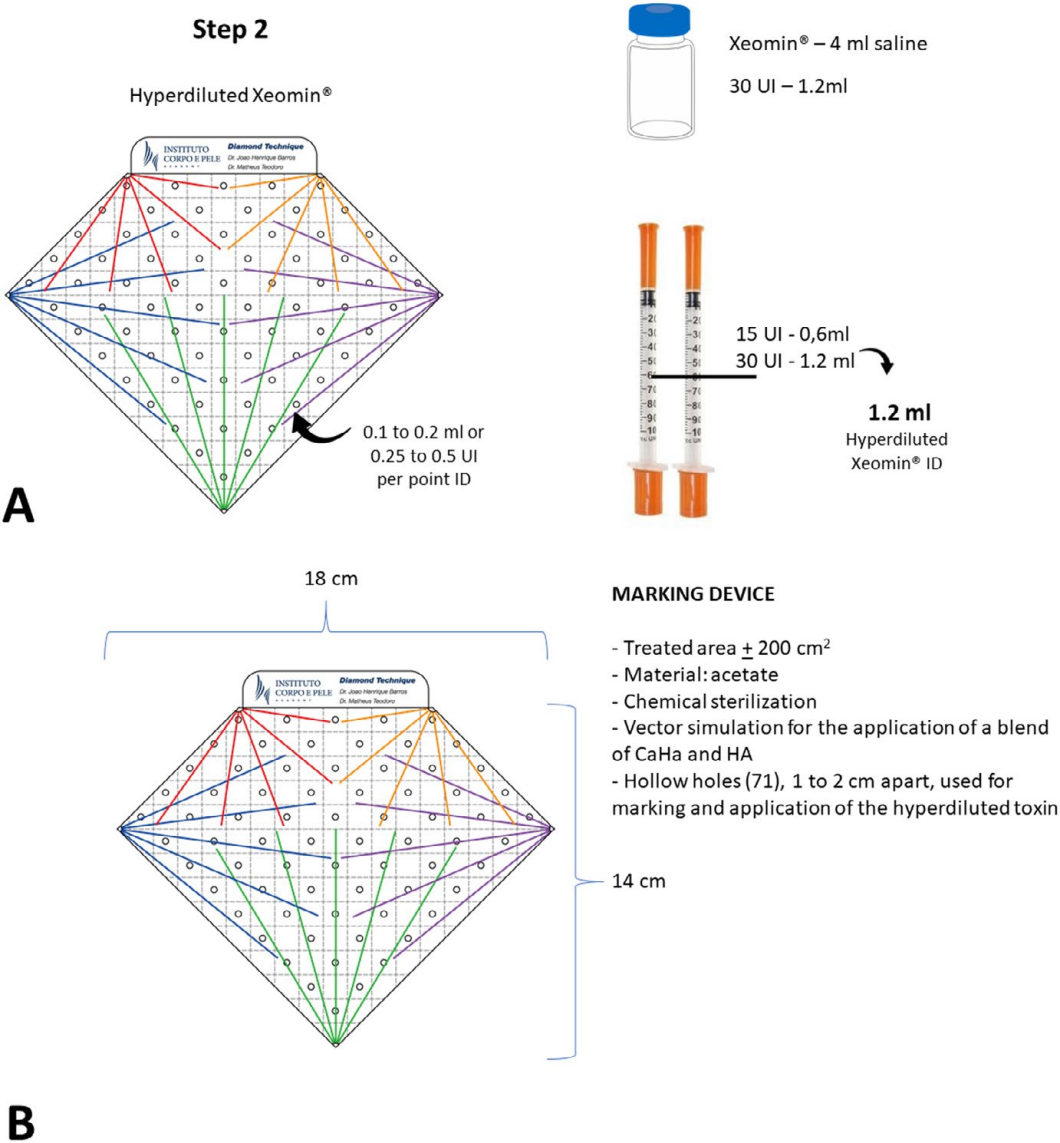
(A) Schematic representation of the preparation of the hyperdiluted neurotoxin for the treatment. The injections of 1–2 UI should be administered intradermally at the markings, 1–2 cm apart, set by the perforations of the “diamond” device. (B) Detailed characteristics of the marking diamond‐shaped device.

The second step comprises the preparation of the neurotoxin for the treatment. One vial of 100 U of INCO (Xeomin, Merz Pharmaceuticals) is diluted with 4 mL of 0.9% saline solution (hyperdilution 0.25 UI/0.1 mL). One aliquot of 1.2 mL (30 U) should be allocated for the treatment (Figure 2A).

**FIGURE 3:**
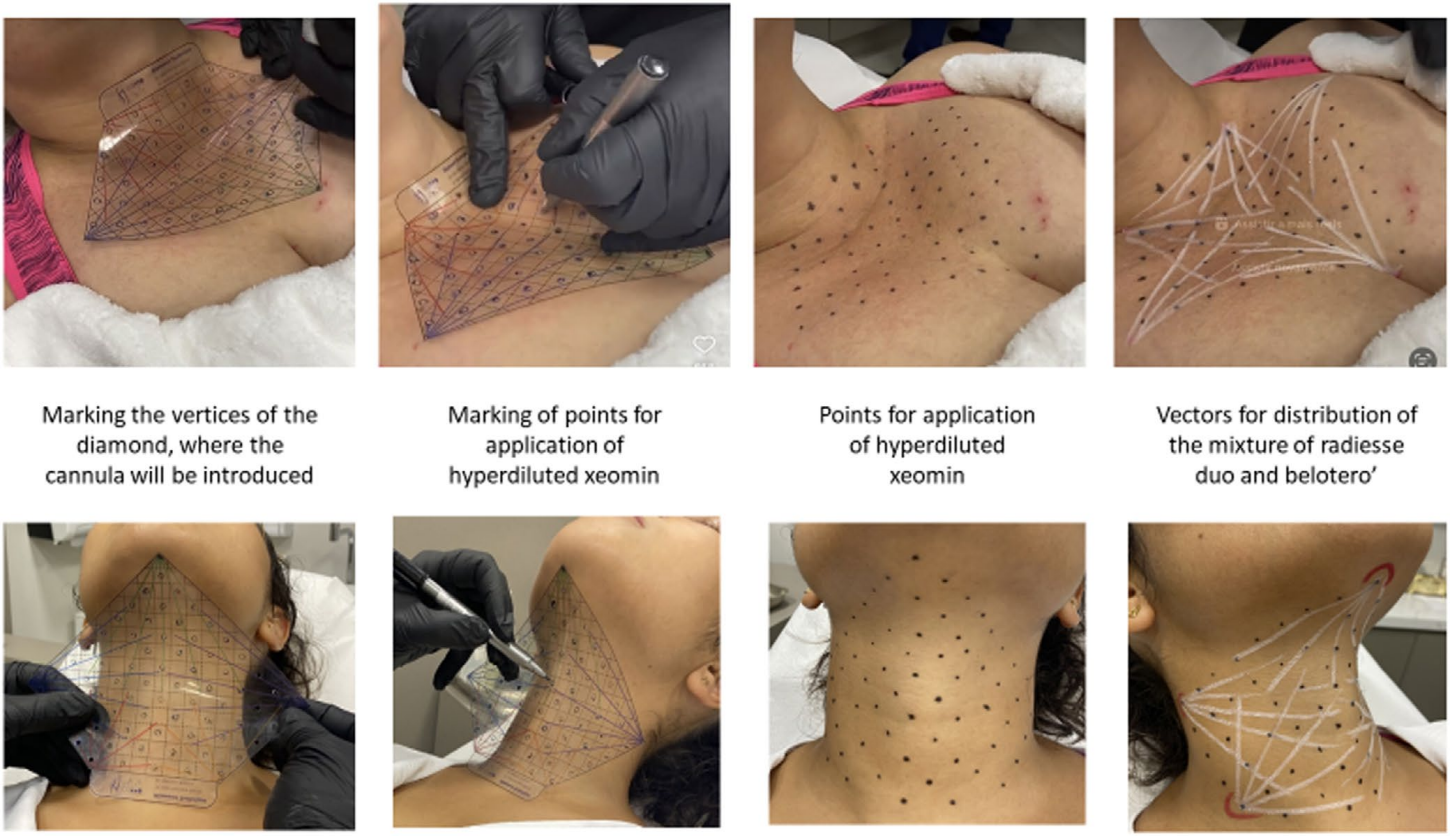
Demonstration of the skin markings on the skin vectors (HA and CaHA) and the injection points for the hyperdiluted INCO. For the neck, the upper vertex of the diamond‐shaped device, used in a mirrored manner, is positioned at the level of the mental spine, while its two lower vertices are directed toward the upper edge of the clavicles. For the décolleté, the two upper vertices of the diamond are positioned at the level of the upper edge of the clavicles, while its lower vertex is directed toward the intermammary sulcus.

The Diamond technique uses a diamond‐shaped acetate device (Figures 2B and 3) with strategic hexagonal markings of specific measurements to guide the triple treatment (CaHA, HA, and INCO). The device is available for practitioners under contact with the corresponding author.

The orientation of the orifices of the diamond is based on the recommendations for fillers, biostimulators, and neurotoxins in this region; moreover, it delimitates the area of treatment [8, 19, 20]. Each vertex of the diamond represents an entry point for the cannula, from which five colored vectors emerge. For each vertex, 1.5 mL of the solution described in Step 1 (Figure 1B) is allocated, consequently, 0.3 mL on each vector, and administered through retroinjection in a subdermal plane using a 22G × 70 mm cannula. The device has perforations along its length, spaced 1–2 cm apart, designated as marking points where hyperdiluted (0.1–0.2 mL) INCO, described in Step 2, is applied intradermally.

For the décolleté, use standard positioning of the diamond‐shaped device to mark the five vertices (representing the points of introduction of the cannula, aiming to cover the entire target treatment area); vectorial markings (five vectors for each vertex) indicate strategic points for the application of hyperdiluted botulinum toxin (71 punctures). The vectors show the area where the highly diluted solution (containing CaHa and HA) should be dispersed, and the hollow points mark the sites where the hiperdiluted INCO should be applied.

For the neck, the device is positioned upside down, with the inferior apex directed superiorly (Figure 3); marking of the five vertices (representing the points of introduction of the cannula); vectorial marking (five vectors for each vertex, surveying the entire target area to be treated); marking of strategic points for the application of hyperdiluted botulinum toxin (71 punctures).

The treatment sequence involves first, the application of the CaHA + HA mixture (0.3 mL of the mixture per vector, totaling 1.5 mL per cannula insertion point). Then, immediately after, with the area already anesthetized, apply 0.1–0.2 mL of hyperdiluted toxin intradermally at each marked point.

We selected four female routine patients to demonstrate the technique, with two categorized as grade II and two as grade III according to the Merz scale of neck laxity at rest [22]. The participants were asked to rate the results using a 5‐point subjective satisfaction scale: worse than before, “below expectations,” “average results,” “good results,” and “beyond expectations.”

All patients were instructed to avoid physical exercise and perform active circular massage twice a day for the 7 days following the procedure.

## 3 Results

The participants were four women, aged between 55 and 63 years, with no previous treatments performed on the neck and décolleté regions.

**FIGURE 4:**
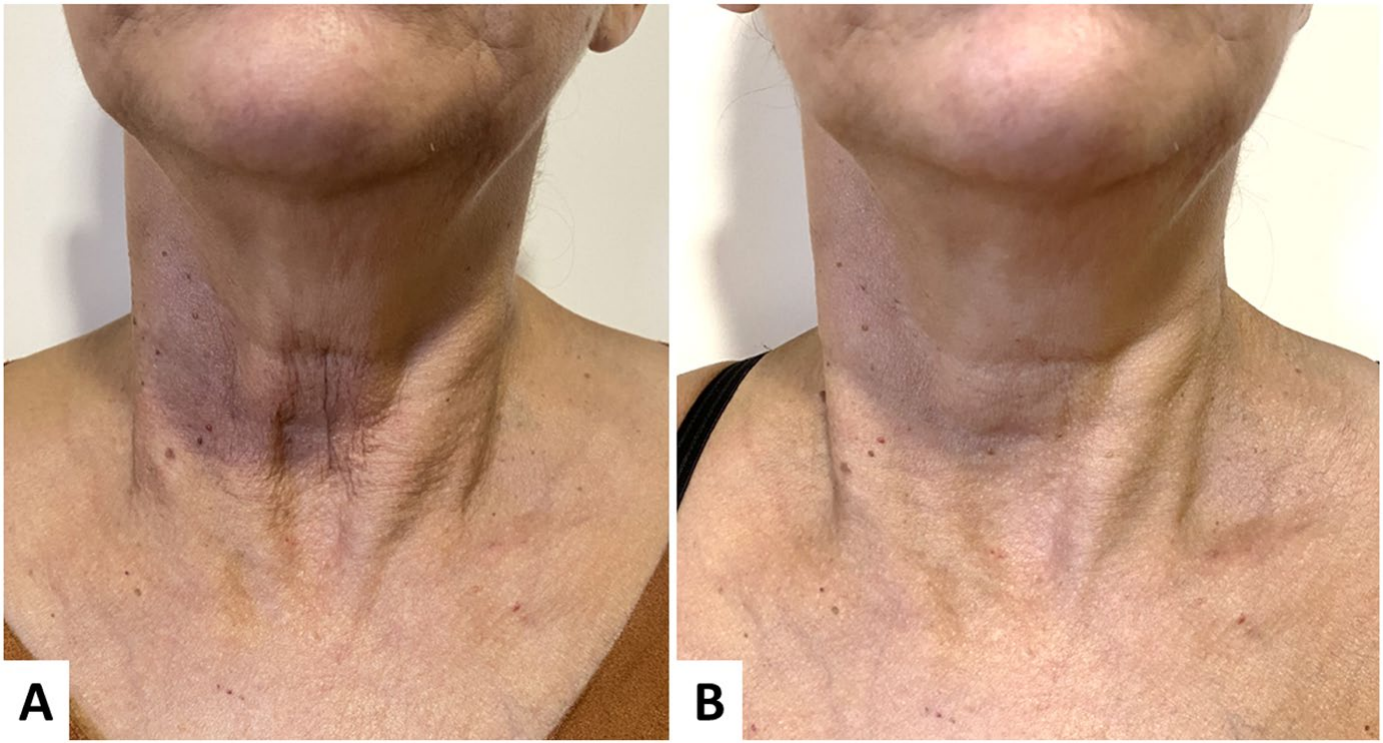
Diamond technique for the neck and décolleté. (A) Before treatment. (B) 90 days after treatment. There was an overall improvement in skin laxity, static wrinkles, and skin quality. In the center‐medial region (lower portion), there is a noticeable smoothing of perpendicular wrinkles and a revitalized appearance of the skin.

**FIGURE 5:**
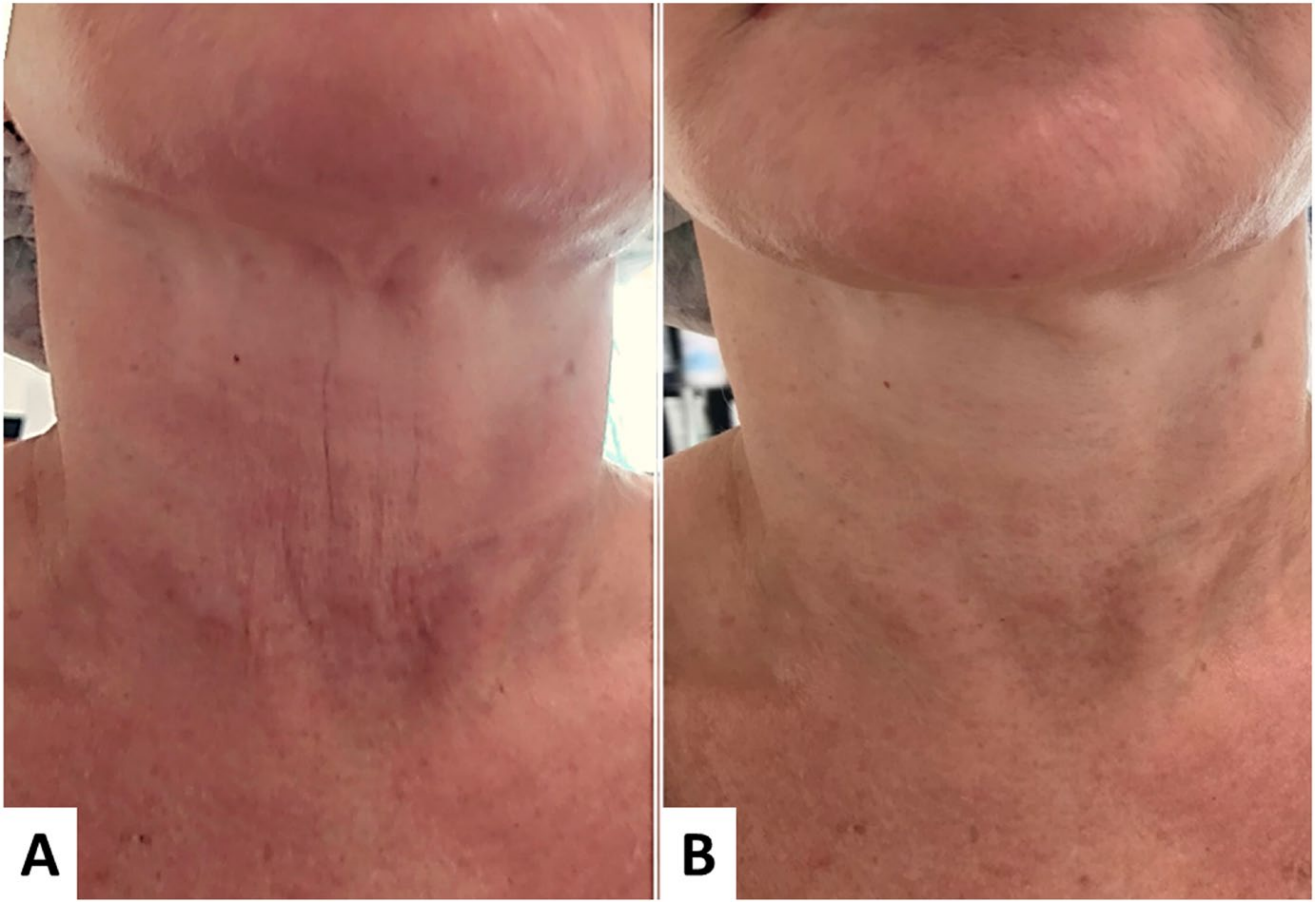
Diamond technique for the neck. (A) Before treatment. (B) 90 days after treatment. There was dermal densification, cutaneous restructuring with improvement in quality and hydration, as well as tissue repositioning, resulting in neck rejuvenation and beautification.

**FIGURE 6:**
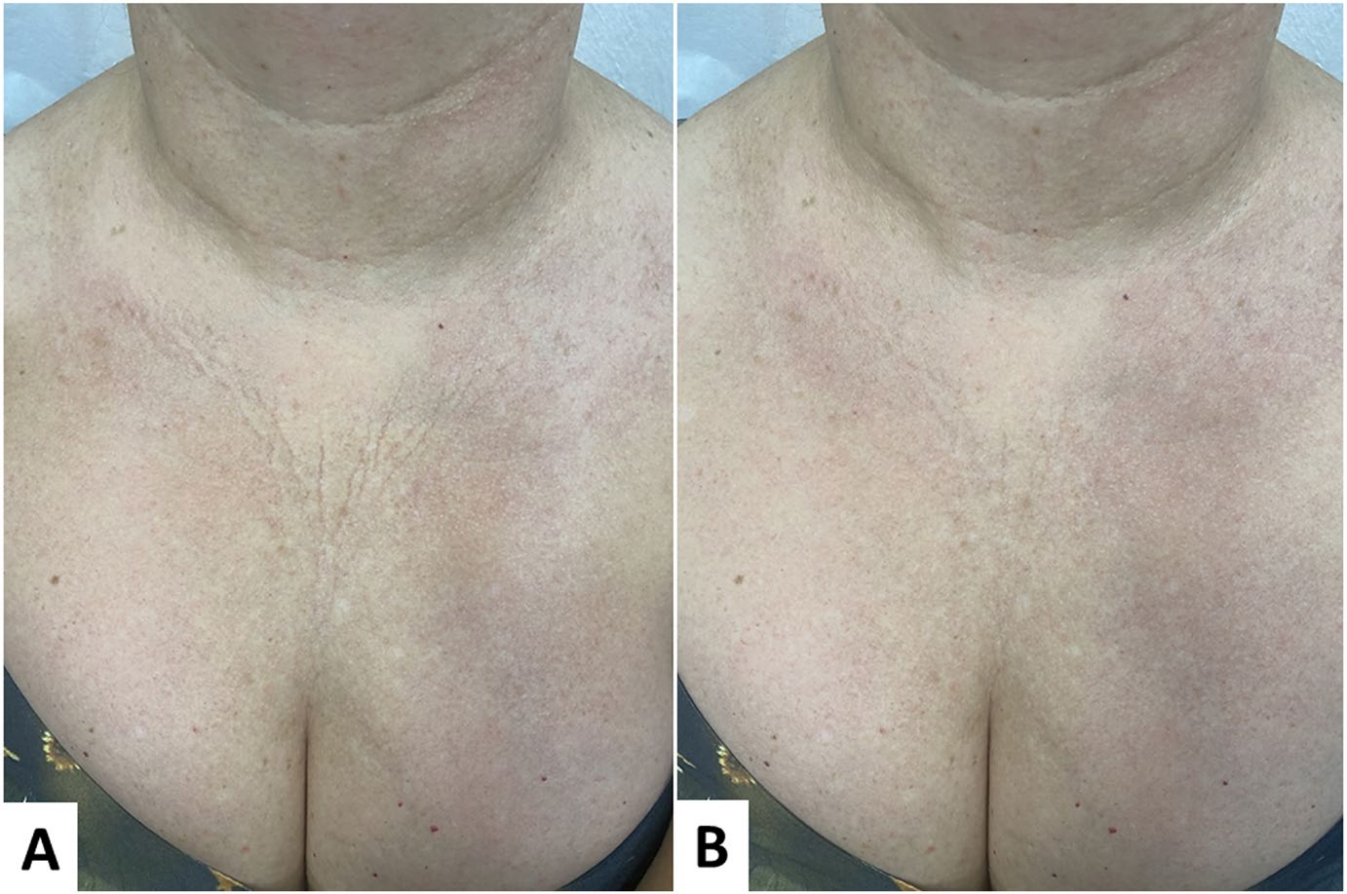
Diamond technique for the décolleté. (A) Before treatment. (B) 85 days after treatment. Smoothing of static wrinkles as well as overall improvement in skin quality. Dermal densification and cutaneous restructuring, promoting local rejuvenation.

**FIGURE 7:**
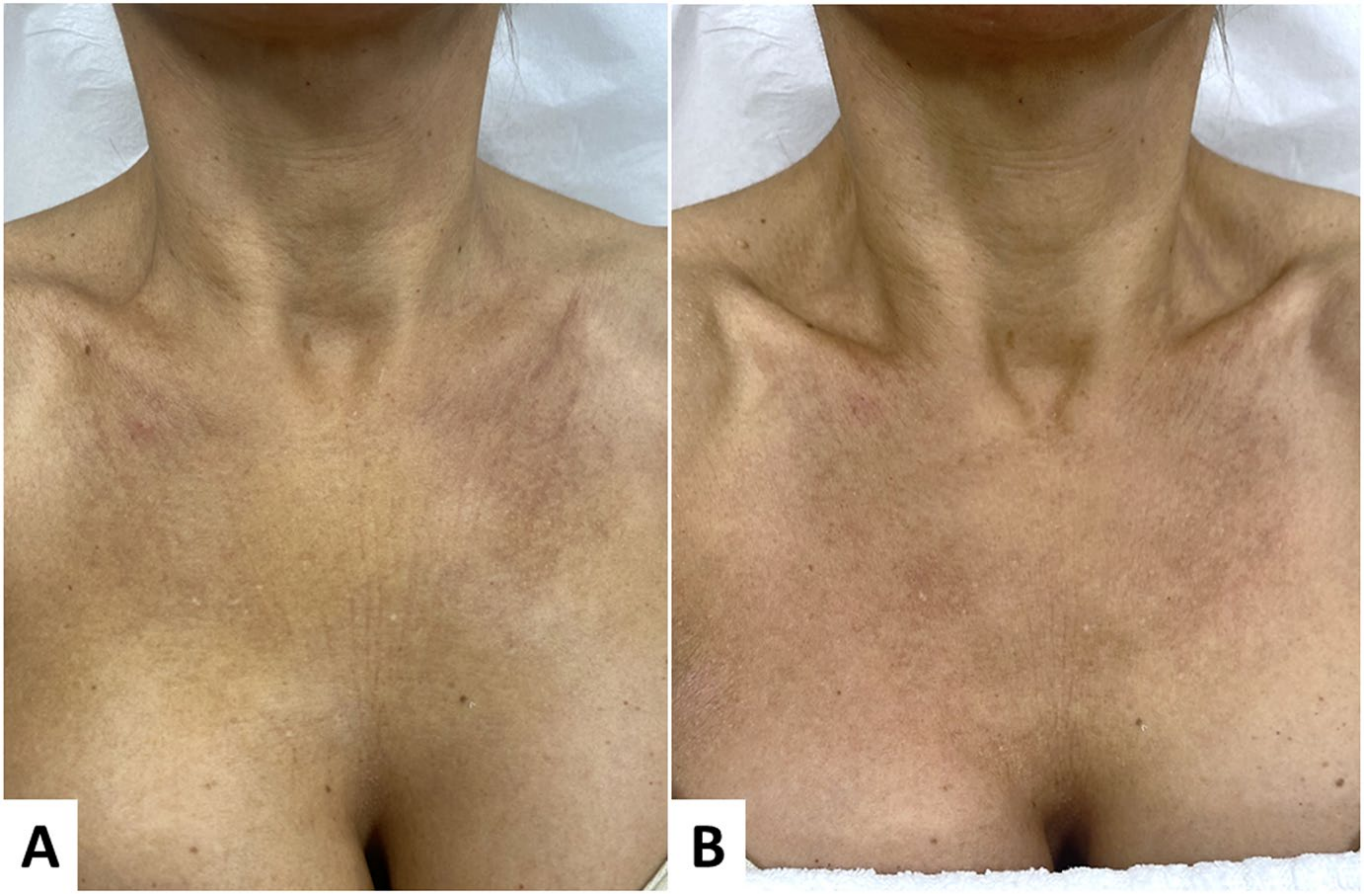
Diamond technique for the décolleté. (A) Before treatment. (B) 95 days after treatment. Global improvement of the skin with rejuvenation and beautification, including the smoothing of static wrinkles, dermal densification, and tissue restructuring.

The treatments are illustrated below as examples of the results achieved with the diamond technique. All the cases presented an overall improvement in skin laxity, static wrinkles, and skin quality following 90 days after the treatment (Figures 4, 5, 6, 7). Two participants reported “good results,” and two reported results “beyond expectations.”

The procedures were well‐tolerated, and subsequent pain was negligible. No adverse effects were observed except for transient edema and bruising. Furthermore, there was no requirement for touch‐ups or retreatment. All participants expressed their willingness to undergo the procedure again if necessary.

## 4 Discussion

Neck and décolleté rejuvenation should be promoted by a combination of measures aimed to revert the aging process, such as environmental exposure (e.g., ultraviolet radiation, air pollution), poor posture, pigmentary alterations, surface changes, skin thinning, and the activity of the platysma [23].

Combined treatments, including technologies, neurotoxins, biostimulators, dermal fillers, peelings, and topical drugs, are recommended to address the various dimensions of neck and décolleté aging [20, 24]. Combining CaHA, HA, and INCO can restore firmness, smoothness, and hydration and reduce platysmal bands.

Minimally invasive (nonsurgical) cosmetic procedures (e.g., neurotoxins, fillers, and biostimulators) have a low rate of adverse effects and short downtime, resulting in a natural appearance [25]. Furthermore, these procedures can be repeated periodically according to individual cosmetic demands [26].

Here, we present a minimally invasive technique that combines three products to simultaneously address the aging signs of the neck and décolleté by using a diamond‐shaped device to guide the skin markings for the procedure. The “diamond” device brings agility to the marking process and technical reasoning, without compromising the individualization of each treatment. In addition, the simple and well‐standardized technique eases medical training and increases reproducibility. Moreover, the technique can be tailored as the composition of Step 1, involving more CaHA and/or HA, can be changed according to the needs and customization of each treatment and patient. Consequently, highly predictable clinical results and patient satisfaction are possible when the Diamond technique is strictly followed.

Complications, such as pain, swelling, erythema, and bruising, associated with CaHA injections are infrequent, with the majority being temporary. Vascular complications are possible in this region due to the plane of injection. Migration is not typically observed; however, erythematous nodules and granulomas have been reported with superficial CaHA injections, particularly in dynamic areas, such as the neck [14, 27, 28, 29].

For rejuvenation, HA‐based injectable fillers are currently the gold standard for restoring volume, leading to improved skin texture and hydration. Diluted HA is also well‐tolerated with mild and transitory adverse effects (e.g., bruising, edema); nodules, infection, and granulomatous reactions are rare, especially if used in low volumes [28, 30].

CaHA acts as a biostimulator, promoting collagen and elastin production, while HA provides immediate hydration, restoration, and volumization due to its water‐retaining properties. Together, they offer both immediate and long‐term benefits: HA enhances skin hydration and elasticity, while CaHA stimulates deeper regenerative processes. This synergy results in improved skin texture, firmness, and overall skin rejuvenation [11, 12, 13, 15, 16].

IncobotulinumtoxinA (INCO) is a highly purified botulinum toxin without other unnecessary clostridial proteins. Treatments for neck and décolleté rejuvenation have been reported to reduce the appearance of horizontal lines and soften the platysmal bands; in the décolletage, INCO can smooth wrinkles caused by sun damage and natural aging, providing a rejuvenated appearance [17, 18].

Neurotoxins are generally well‐tolerated in the neck and décolleté regions. Multiple points of application of a higher diluted neurotoxin are advisable for these areas to minimize adverse effects and achieve optimal results [17, 31]. Bruising, swelling, and pain at the injection site are common, although temporary. Although neutralizing antibodies are rare following aesthetic procedures, especially when low doses are used, there is no information on immunogenicity when combined treatments are performed on the same day [32]. One of the primary concerns is the inadvertent spread of the toxin to adjacent muscles, especially the sternocleidomastoid and some swallowing muscles inserted in the hyoid bone [33, 34].

As platysma is a broad, thin sheet‐like muscle, intramuscular injections of neurotoxins can lead to a larger zone of diffusion, potentially affecting unintended muscles and leading to unwanted side effects. A thorough understanding of the anatomy and a low‐volume intradermal injection is crucial to avoid such complications [35, 36, 37].

Several protocols involve the simultaneous injection of diluted botulinum toxin with HA and/or CaHA, demonstrating the additional benefits of this combination for addressing various aging aspects in the neck region [17, 19, 38, 39, 40]. The admixing of CaHA and hyperdiluted INCO for application with a cannula in the neck (Relax and Firmness technique) resulted in high early satisfaction among participants, with no reported adverse effects [8]. The simultaneous injection of CaHA, HA, and INCO in the same solution lead to improvement in 93% of the patients [19]. Hybrid solutions (e.g., CaHA and HA in the same syringe) allow for the simultaneous benefits of each product in a single session, resulting in highly satisfactory outcomes [21]. Moreover, the premixing of these products before injection was found to be safe in a 12‐month follow‐up of 134 patients [29].

This report aimed to introduce a novel protocol for triple and simultaneous treatment targeting the neck and décolleté regions. However, it is important to note that this report solely outlines the protocol and lacks the controlled, blinded, and prospective studies necessary to validate efficacy and cost‐effectiveness in comparison with existing approaches for these areas [41].

The authors treated more than 50 patients using this technique over 5 years without severe adverse effects, which reinforces its safety. Nevertheless, extensive series with long‐term follow‐up and objective measurements are essential to establish the technique's safety and effectiveness. Future research endeavors should focus on conducting rigorous comparative studies to provide comprehensive data and insights into maximizing the outcomes of this treatment approach for the neck and décolleté regions.

## 5 Conclusion

The Diamond technique employs a blend of diluted CaHA and HA along with INCO to target different aspects of neck and décolleté aging simultaneously. The procedure is facilitated by a specialized plastic device shaped like a “diamond,” aiding in precise skin markings for injections. This minimally invasive approach not only saves time but also ensures excellent tolerance, resulting in high patient satisfaction and consistent, predictable outcomes.

## Ethics Statement

Institutional Review Board appraisal was not required or sought as this report was not part of a prospective or systematic investigation of biostimulator treatment, nor identifiable images were presented. Moreover, it followed general principles for routine CaHA, HA and INCO injection. Institutional approval was not required to publish these unidentified cases. The authors ensure that participants' rights are protected. All procedures in studies involving human participants were performed in accordance with the Declaration of Helsinki (as revised in 2013).

## Consent

All patients provided informed consent for the case details and images to be published in this report.

## Conflicts of Interest

Dr. Matheus dos Santos Teodoro and Dr. Ana Lúcia Gonzaga da Cunha are medical speakers for Merz. Dr. João Henrique da Fonseca Armada Barros declares no conflicts of interest.

## Data Availability

The data that support the findings of this study are available on request from the corresponding author. The data are not publicly available due to privacy or ethical restrictions.
